# DIY Universal Fraction Collector

**DOI:** 10.1021/acs.analchem.1c01519

**Published:** 2021-06-25

**Authors:** David Díaz, Ana de la Iglesia, Francisco Barreto, Ricardo Borges

**Affiliations:** ^†^Unidad de Farmacología, Facultad de Medicina and ^‡^Servicio de Electrónica, Universidad de La Laguna, E-38200 La Laguna, Tenerife, Spain

## Abstract

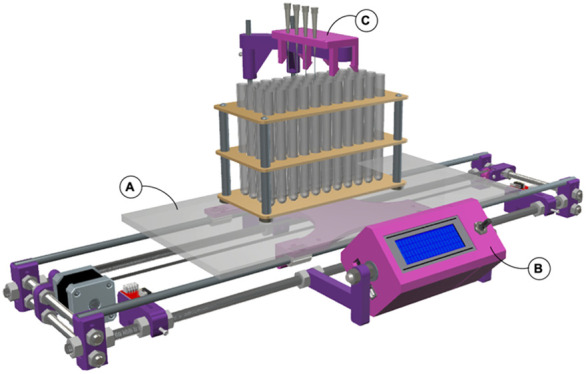

Fraction collectors are common pieces of equipment that are essential for the activity of many biochemistry, pharmacology, and drug discovery laboratories. However, these devices are not very versatile when it comes to tailoring them to specific needs, such as different size collection tubes, sequences of tube exchange, or parallel collection. In addition, these systems are relatively expensive, especially for small laboratories or for those in less developed countries. The emergence of 3D printers and the availability of cheap, popular electronic control devices are changing the way laboratory equipment can be made and designed. Here, we describe how to build your own fraction collector, indicating all the elements and providing the full instructions needed to make a fraction collector that can be adapted to almost any kind of rack and tubes (3D files, the parts required, the electronic circuits, and the software). This device can be used in complex protocols, adapted to liquid chromatography and for parallel collection from perfused tissues. The total cost of the whole device is around €100.

The arrival of open-source platforms for the programming of hardware and the popularization of 3D printers has enabled practical devices to be designed, produced, and incorporated into the analytical pipelines of many laboratories. This opportunity is especially interesting when considering initial prototypes, research groups with limited income or funding, and/or student projects.

Fraction collectors are devices commonly found in life science laboratories, usually coupled to chromatography or perfusion systems. However, it is often difficult to adapt these machines to satisfy all the needs for sample collection and to customize them to specific experimental designs (e.g., different tube sizes or special timing-event patterns for sampling). In addition, there are few commercially available devices that are capable of collecting several samples simultaneously, in parallel. Indeed, this latter feature has inspired us to design and manufacture a very affordable and versatile fraction collector, which can be adapted to virtually all laboratory requirements. In our laboratory, this device is currently used to collect the fluid emanating from superfused adrenal chromaffin cells.^1,2^ Here, we provide a full description of our low-cost DIY fraction collector, built from materials available at any local hardware and electronics shop and using a 3D printer.

## Experimental Section

### Design and Implementation

**Figure 1:**
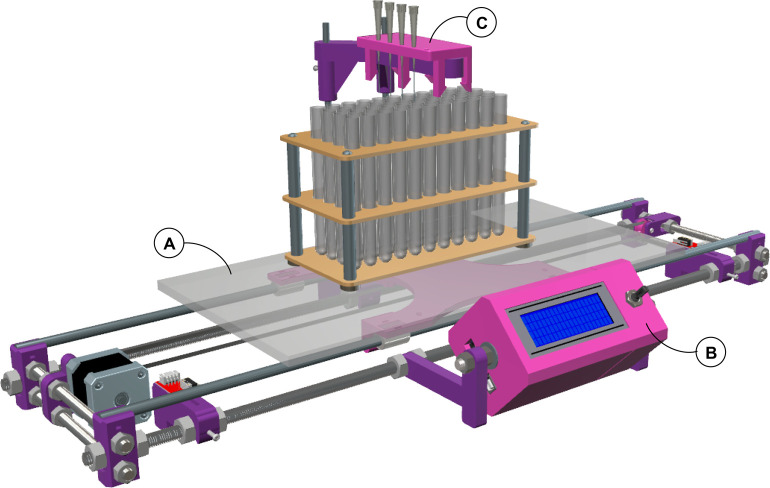
3D overview of the universal fraction collector. Images: (**A**) Mobile platform. The use of a flat platform allows almost all kinds of tube racks to be accommodated, from 1.5 mL Eppendorf-tubes to large (≈50 mL) test tubes. This apparatus has enough precision to allow fractions to be collected in 96-well plates. Also, we usually place an ice tray on the platform to cool the tubes and preserve the samples collected. For clarity, this ice tray is not shown. (**B**) Programmable electronic system. (**C**) Dripper.

Initially, the fraction collector (Figure 1) was designed to satisfy our specific experimental needs. However, this is a very versatile device, and it can be used in a wide variety of experiments and for different applications that require sample collection, even those that require pauses between the collection of the samples.

The collector is essentially composed of three parts: (i) a mobile platform that accommodates the tube racks (Figure 1, part A); (ii) an electronic system to control and program the platform’s movement (Figure 1, part B); and (iii) a dripper that is designed for parallel collection from multiple drop dispensers (Figure 1, part C). In terms of its operation, the universal fraction collector is designed to offer four different modes: return, manual, auto, and calibration. However, it is also possible to reprogram the system to add new modes or modify existing ones. In the return mode, the system forces the mobile platform to return automatically to its initial position, where it stops after pressing the end-stop switch. If the manual mode is selected, the mobile platform moves forward or backward one step when the user moves the encoder once clockwise or anticlockwise turn, which makes this mode useful to position the platform anywhere along its path. Conversely, in auto mode, the system awaits an external TTL (+5 V) pulse signal, and the mobile platform moves forward one step from its current position when a pulse is detected. This mode allows the device to be synchronized with other laboratory instruments. Finally, the calibration mode is designed to configure the step length (in millimeters) during the operation mode, and therefore, the step length can be set to match the separation between the collecting tubes. Alternatively, this mode can be used to place the dripper between two tubes when collection is not desired. The user can access the different modes, calibrate the system, and drive the mobile platform manually using the LCD screen and the rotary encoder that has an integrated push-button on the Arduino screen.

Although the current firmware was set for a minimum step of 1 mm, this open-source file can be easily modified to a step of 0.1 mm for very small racks, including 96-well plates.

To construct the device, we combined 3D printed plastic pieces with metal parts available in any local hardware store, such as rods, threaded rods, nuts, and washers (a full description of all the metal parts required can be found in the Supporting Information). We designed our own 3D printed plastic pieces, and we used others taken from the popular BQ Prusa i3 Hephestos 3D printer web repository. All these pieces were printed with PLA, although it is possible to use other materials such as ABS or nylon. These files are available for free download in STL format at http://rborges.webs.ull.es, and a full description of each plastic piece is provided in the Supporting Information.

#### Mobile Platform

**Figure 2:**
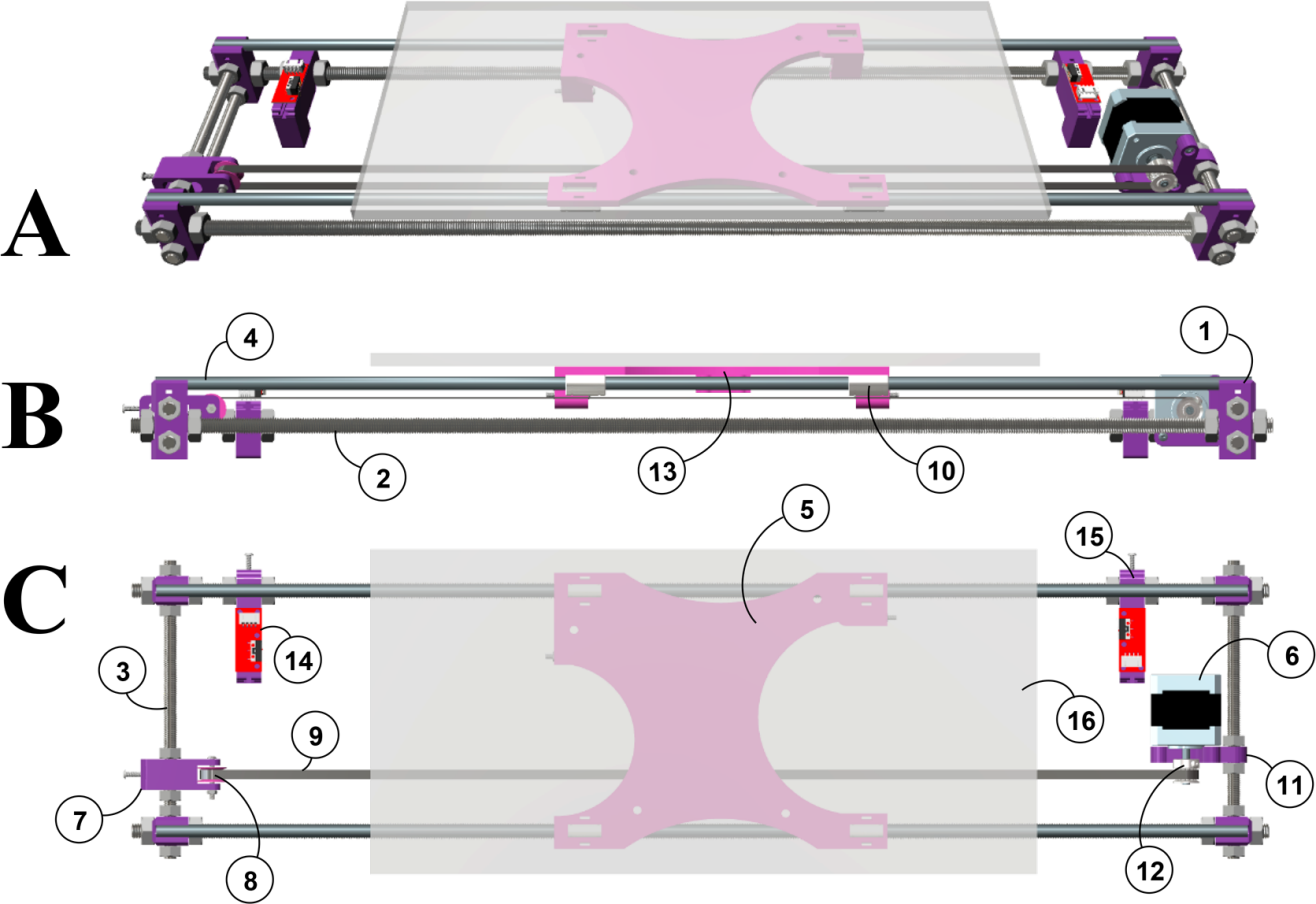
3D views of the mobile platform. Images: (**A**) Front view perspective. (**B**) Orthographic front view. (**C**) Orthographic top view. Items: (**1**) 3D printed corner; (**2**) metal Ø 10 mm threaded rod (50 cm long); (**3**) metal Ø 8 mm threaded rods (20 cm long); (**4**) metal Ø 8 mm rods (50 cm long); (**5**) 3D printed carriage; (**6**) 17HS8401 4-lead Nema17 stepper motor; (**7**) 3D printed belt tensor; (**8**) 3D printed pulley with 623ZZ bearing placed inside; (**9**) GT2-6 mm toothed belt; (**10**) LM8UU linear bearing; (**11**) 3D printed motor holder; (**12**) GT2 20 tooth/shaft Ø 5 mm aluminum gear; (**13**) 3D printed belt clamp; (**14**) MakerBot end-stop switch; (**15**) 3D printed holder end-stop switches; (**16**) methacrylate plate (45 × 20 × 1 cm).

We have built a one-axis lineal platform capable of accommodating any tube rack up to 35–40 cm in length (Figure 2). To assemble the base, we used 4 plastic corners, 6 threaded metal rods (two Ø 10 mm, 50 cm long and four Ø 8 mm, 20 cm long), and 2 metal rods (Ø 8 mm, 50 cm long: Figure 2, items 1 to 4). These pieces are fixed in place using nuts, washers, and plastic bridles. Although the dimensions of the system are sufficient for our needs, its size can be adjusted to the users’ demands. Overall, this frame provides a solid, vibration-free base for the other parts of the fraction collector.

The base of the structure has a plastic carriage (Figure 2, item 5) that can be moved in both directions by a stepper motor that drives a toothed belt attached to a pulley via a belt-tensor (Figure 2, items 6 to 9). The carriage is mounted on four linear bearings to ensure a smooth displacement (Figure 2, item 10), each installed on the Ø 8 mm rods. Plastic bridles are used to attach the carriage to the bearings. The belt-tensor and stepper motor holder (Figure 2, item 11) are also mounted on the Ø 8 mm threaded rods, one on each side of the structure. To complete the mechanism, the toothed belt passes through the belt-tensor pulley and the stepper motor gear (Figure 2, item 12), and it is then attached to the underside of the carriage using a plastic belt holder (Figure 2, item 13). To limit carriage displacement, two electronic end-stop switches (Figure 2, item 14) are fixed to the same Ø 10 mm threaded rod using custom plastic pieces (Figure 2, item 15). Two Ø 3 mm screws at the back of these pieces allow the user to set the desired position along the stainless-steel rod.

Finally, a rectangular methacrylate plate (45 cm long × 20 cm wide × 1 cm thick: Figure 2, item 16) is screwed onto the carriage, allowing it to support any tube rack placed on a ice tray to cool the samples collected. This plate was designed to satisfy our needs, but it can be tailored to accommodate almost any kind of tube rack (A template to drill the holes used to screw the rack onto the carriage is provided in the Supporting Information.). Silicone bumpers under the tube racks (≈Ø 1 cm) provide a stable surface, as recommended (see the Supporting Information for more details about the mobile platform assembly).

#### Programmable Electronics System

**Figure 3:**
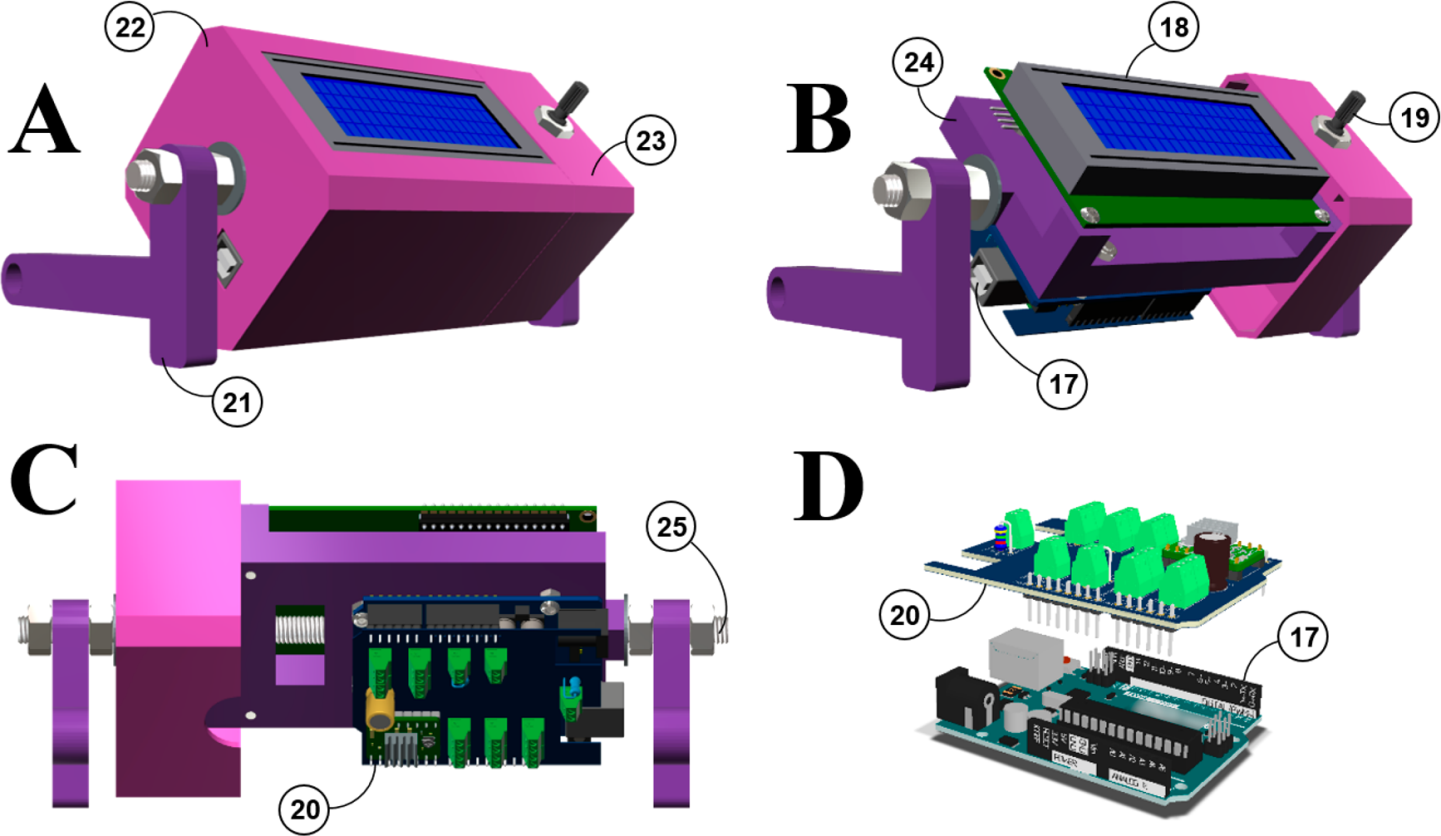
3D views of the programmable electronic system. Images: (**A**) Side view. (**B**) Side view without the case. (**C**) Orthographic rear view without the case. (**D**) Assembly of the Arduino UNO and custom expansion board. Items: (**17**) Arduino UNO board; (**18**) LCD Module 2004A with I2C PCF8574T board adapter; (**19**) EC11 Series Rotary encoder with push-button switch; (**20**) custom expansion board; (**21**) 3D printed foot for the electronic system’s case; (**22**) 3D printed case for the electronic system, part B; (**23**) 3D printed case for the electronic system, part A; (**24**) 3D printed holder for the electronic devices; (**25**) metal Ø 8 mm threaded rod (20 cm long) with nuts.

We have used the popular Arduino UNO board to implement the core of our electronic system (Figure 3, item 17), which controls the stepper motor and reads the status of end-stop switches. In addition, the system includes a LCD display to show the configuration menus and to monitor the status of the fraction collector (Figure 3, item 18). To navigate through the system’s options and drive the mobile platform, a rotary encoder with an integrated push-button is used (Figure 3, item 19). In general, our fraction collector is a standalone apparatus that can work as an independent machine. Nevertheless, mobile platform movements can also be triggered from an external TTL signal to synchronize displacement using local computers, chromatography controllers, or other laboratory instruments. The Arduino UNO program was created using Arduino IDE 1.8.5 software, and all the program files are available for free download at http://rborges.webs.ull.es.

We use our own expansion board to wire the Arduino UNO board to the other electronic devices (Figure 3, item 20), which includes plug connectors to achieve easy and reliable connections. This board also has the stepper motor power-driver installed (see the Supporting Information for a detailed description of our expansion board, including the schemes and wiring maps). GERBER files are available for free download at http://rborges.webs.ull.es.

To accommodate all the electronics, we designed a compact plastic box (Figure 3, items 21 to 25), and all the wires are fixed to the frame using spiral wraps. Finally, a 9 V/1A DC power supply is directly connected to the Arduino UNO board power connector to power the entire system.

#### Dropper

**Figure 4:**
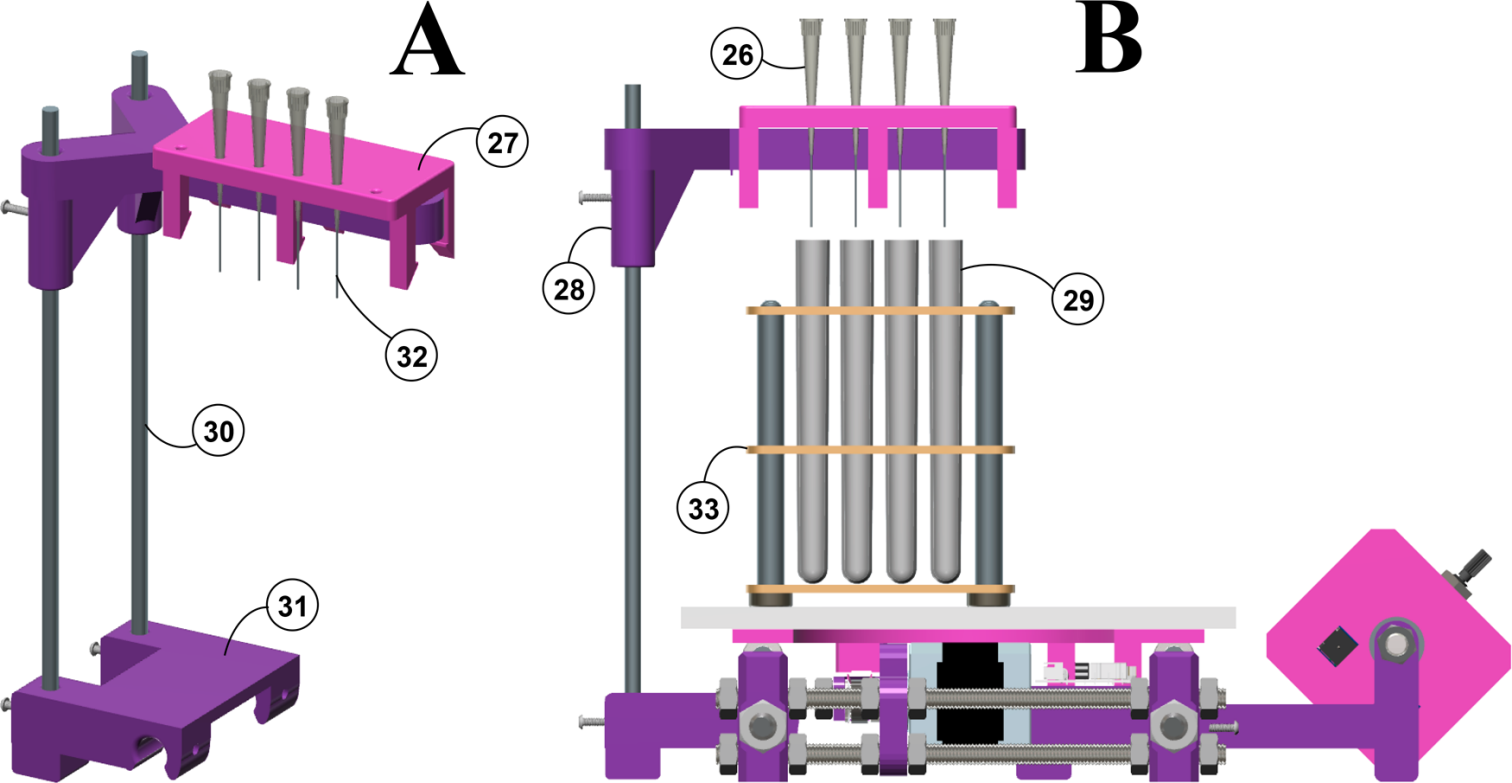
3D views of dripper. Images: (**A**) Side view. (**B**) Orthographic side view of the fraction collector. Items: (**26**) droppers; (**27**) 3D printed dropper holder; (**28**) 3D printed beam; (**29**) tubes; (**30**) metal Ø 6 mm rod (25 cm long); (**31**) 3D printed stand; (**32**) stainless-steel tubing; (**33**) tube rack.

Our dropper is conceived for sample collection, using up to four parallel droppers. In addition, it is designed to be able to adjust the distance between the drop dispensers and tube racks of different heights. To fix the droppers (Figure 4, item 26), we use a custom plastic holder (Figure 4, item 27) that was designed to position the droppers in a row, 1.5 cm apart. This distance matches the separation between the tubes in our racks. Nevertheless, it is not difficult to design/print a dropper-holder that is compatible with other tube rack dimensions.

The plastic beam that supports the holder is placed perpendicular to the forward direction of the mobile platform (Figure 4, item 28). Thus, the row of droppers is perpendicular to the forward direction of the tubes (Figure 4, item 29). The beam height can be adjusted using two vertical stainless-steel bars (Ø 6 mm and 25 cm long: Figure 4, item 30) to adjust the distance from the top of the tube. Two Ø 5 mm screws are situated at the back of the beam to block it. Finally, the two vertical bars are anchored to a plastic stand (Figure 4, item 31) that is fixed to the base structure by nuts (see the Supporting Information for more details about the dropper mounting).

## Results and Discussion

This device was built to fulfill our specific needs, which seem to be common to those of many laboratories. We needed a versatile fraction collector to achieve multiple parallel collections of samples ranging from 0.5 to 20 mL. We also required intermittent collections (i.e., a run of three samples, a pause, and another sampling run), for which the system must allow sample dropping in between the two adjacent tubes. We currently couple this device to a four-channel potentiostat (CANSTAT-4)^1,3^ for the simultaneous recording of catecholamine release from adrenal chromaffin cells and to collect samples from the effluent. For us, the advantage of using this device is the reproducibility of the results, and once programmed and synchronized, it is no longer affected by operator fatigue when changing the tubes as the experiment proceeds (over an hour).

Here, we describe our routine sample collection from perifused chromaffin cells or from perfused rat adrenal glands, initially moving the platform to its initial position using the return mode option. We then fill a 20 × 6 rack with 5 mL tubes and place it on the mobile platform. As we analyzed catecholamines, we usually add perchloric acid to all the tubes to a final concentration of 0.05 N, and the waste tray was filled with ice to maintain the samples chilled. Once the perfusion system is operative, we manually adjust the height of the dropper and align it to the tubes. The first line of tubes should be just one step before the position below the droppers. The calibration mode was then selected, and we entered the desired value for the platform step in millimeters. We usually program the step distance value as half of the distance between the tubes. This allows us to rule out unwanted samples because they drip between the tubes directly onto the waste tray. Consequently, for consecutive collection, we program two steps on the encoder (in manual mode) or we execute two consecutive TTL pulses from the external device (in auto mode). We then use the manual mode to ensure that all the tubes are in the correct position at each step of the platform. If not, the tube rack placement on the platform must be readjusted or the distance value verified. It is advisible to set the starting position one step before the first collecting tube to drop all the initial material onto the waste tray.

A standard experiment starts with a 10 min stabilization period in which the dropper is placed over the first tube, and we then collect 1 min samples prior to stimulating the cells. This is followed by a 1 min stimulus and then 5 min without collection by dropping between tubes. We usually repeat this sequence 10 times, collecting 20 tubes per tissue. As we collect samples from four parallel cell beds, we obtain 80 samples per experiment. Usually, we then analyze catecholamines by HPLC-ED, ATP using a luciferase assay, and protein fragments in Western blots (see the Supporting Information for a video showing the fraction collector in operation).

There are commercial devices that perform similar tasks to our fraction collector, but they are very expensive and take up too much bench room. Our apparatus cost less than €100 to produce, and it occupies just 55 × 35 cm of the bench. Moreover, its construction is fully reconfigurable, and its operation can be reprogrammed to satisfy a wide range of experimental requirements. For these reasons, it is an ideal solution for small and low-income laboratories, including laboratories in developing countries. Building tailoring-made equipment using 3D devices has gained popularity and sophistication. For instance, the possibility of producing an autosampler/fraction collector has been recently reported.^4^

Thanks to the appearance of 3D printers, the availability of parts from local 3D shops and hardware stores, and the versatility of the Arduino ecosystem to build electronic prototypes, this device can be built quite cheaply. The repository at http://rborges.webs.ull.es provides information on all the plastic parts to be printed on any standard 3D printer for free download in an STL format, as well as all the details for the assembly of the electronics system, wiring maps, and the software source code to program the Arduino UNO board. The only aspect that must be mechanized is the working surface that, while it could eventually also be printed, we recommend making it from acrylic material (Perspex methacrylate) as the measurements will largely depend on the user’s needs. We offer a template at the repository with all the positions for drilling. Once all the pieces are on the bench, all the mechanical parts can be mounted in less than 3 h.

Although this device satisfies our requirements for parallel collection, the system described here has a one-dimension displacement as it was conceived for parallel collection from four droppers. This means that the number of samples collected is limited to those accommodated in a single row of the tube rack. To increase this, it would be necessary to implement a second-dimension step-motor arm.

## Conclusion

The fraction collector described here is capable of carrying out tasks that cannot be performed by most commercial, more expensive apparatus, such as cooling samples, the use of a variety of tube racks, multiple simultaneous collections, self-programming, and external control, of course, all at a very affordable price.

## Supporting Information Available

Source files for 3D pieces and Arduino program, electronics schemes, list of materials, and planes for mounting (PDF)
Video of full system working (MOV)

## Supplementary Material

ac1c01519_si_001.pdf

ac1c01519_si_002.mov

## Notes

Supporting research data consisting of Ricardo Borges’ official web page (this also contains a copy of PRUSA repository) and Prusa BQ for this article may be accessed at http://rborges.webs.ull.es.
