# Supplementary material for: DIY Universal Fraction Collector
Source: Anal Chem. 2021 Jun 25;93(27):9314–8. doi: 10.1021/acs.analchem.1c01519 (PMC8929665; doi:10.1021/acs.analchem.1c01519)
Supplement: Supplementary file 1 — ac1c01519_si_001.pdf [file ac1c01519_si_001.pdf]

SUPPORTING INFORMATION

A DIY universal fraction collector

David Díaz[1], Ana de la Iglesia[1], Francisco Barreto[2] and Ricardo Borges[1,]*

[1]Unidad de Farmacología, Facultad de Medicina and [2]Servicio de Electrónica, Universidad de La Laguna, E-38200 La Laguna. Tenerife, Spain.

*Author for correspondence

email: rborges@ull.edu.es

TABLE OF CONTENT

- Catalogue of STL pieces
  - Table S1: Mobile platform
  - Table S2: Control box
  - Table S3: Dropper
- Cost of materials
  - Table S4: Hardware parts
  - Table S5: Electronic device
  - Table S6: Expansion board
- Arduino firmware
  - Table S7: Arduino UNO firmware files
- Mounting guidelines
  - Scheme S1: Expansion board
  - Scheme S2: Expansion board views
  - Scheme S3: Wiring
  - Scheme S4: mechanical assembly
    - Mounting steps.
- Video of the full system working

Catalogue of STL pieces

Table S1: Mobile platform

Fig.	item	Image	Description	Repository file	Amount
2B	1	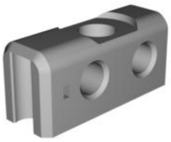	Prusa® corner nut	platform-corner.stl [1]	4
2C	11	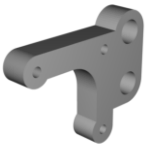	Holder for the step motor	platform-motor holder.stl [1]	1
2C	15	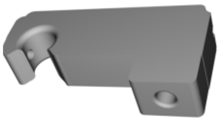	Holder for the end-limit Makerbot® switches	platform-switch holder.stl	2
2C	7	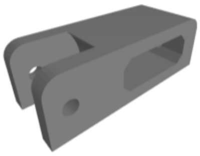	Belt tensor	platform-belt tensor.stl [1]	1
2C	8	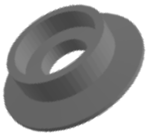	Pulley for belt tensor	platform-belt tensor pulley [1, 2]	2
2C	5	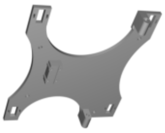	Carriage	platform-carriage.stl	1
2C	13	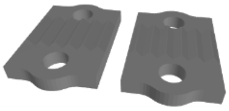	Belt clamps	platform-belt clamp.stl[1]	1
2C	16		Plate drillings	DRL.tiff[3]	1

(1) File obtained from https://www.bq.com/es/support/prusa/support-sheet.
(2) Two pieces are necessary to make the pulley and a Ø 3 mm screw is perfect for axis.
(3) This file contains the diagram for drilling the methacrylate plate.

Table S2: Control box

Fig.	item	Image	Description	Repository file	Amount
3A	21	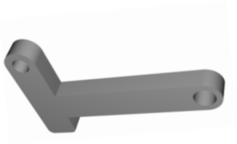	Box holder	controlbox-holder.stl	2
3A	22	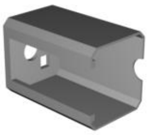	Case Part A	controlbox- case A.stl	1
3A	23	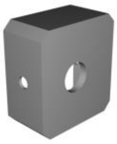	Case Part B	controlbox- case B.stl	1
3B	24	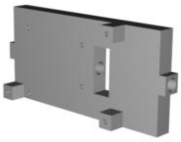	Internal part	controlbox-internal part.stl	1

Table S3: Dropper

Fig.	item	Image	Description	Repository file	Amount
4A	27	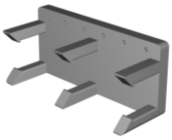	Dropper receptacle	dripper-droppers holder.stl(1)	1
4B	28	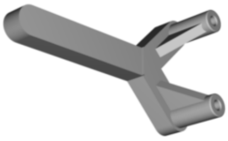	Dropper holder	dripper-beam.stl	1
4A	31	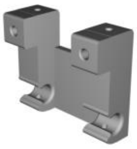	Stand holder for dropping system	dripper-stand.stl	1

(1) This piece is conceived to accommodate six yellow pipette tips.

Cost of the materials

Table S4: Hardware parts

Fig.	Item	Description	Amount	Price (€)
2C	3	Metal Ø-8 mm threated-rod / 200 mm long	4	0.80
2B	4	Metal Ø-8 mm rod / 500 mm long	2	1.50
2B	2	Metal Ø-10 mm threated-rod / 500 mm long	2	1.30
4A	30	Metal Ø-6 mm rod /250 mm long	2	0.60
Not marked		Metal Ø-3 mm nut (for pulley axis)	1	0.08
Not marked		Metal Ø-8 mm nut	20	1.05
Not marked		Metal Ø-10 mm nut	16	1.20
Not marked		Ø-3 mm screws / 20 mm long (for control mount)	7	0.14
Not marked		Ø-5 mm screws / 10 mm long	4	0.10
2C	8	623ZZ bearing (inside pulley)	1	0.30
2C	9	GT2-6 mm rubber belt	1m	1.80
2C	12	GT2 aluminium gear / 20 teeth / shaft Ø-5 mm	1	0.90
2B	10	LM8UU linear bearing	4	4.40
Not displayed		Clear Round Self-Adhesive Rubber Feet Tall Bumpers	8	0.90
2C	16	Methacrylate rectangular piece 200×400×10 mm	1	0.87
			**Total Cost**	**15.94**

Table S5: Electronic device

Fig.	Item	Description	Amount	Price (€)
2C	6	17HS8401 4-lead Nema17 stepper motor	1	13.00
2C	14	Makerbot end-stop switch board	2	0.80
3B	18	LCD Module 2004A + I2C/IIC PCF8574T board adapter	1	12.00
3B	19	Rotary encoder EC11 Series with push-button switch	1	1.50
3D	17	Arduino UNO REV3 board	1	12.00
			**Total Cost**	**39.30**

Table S6: Expansion board

(see fractionCollector-expansionBoardSchematic.pdf file)

Fig. Item	Description	Amount	Price (€)
Not marked	Pololu A4988 Stepper Motor Driver.<br>Schematic part: U1	1	2.00
Not marked	2-way / 2.54 mm pitch PCB terminal block connector<br>Schematic parts: J2, J3, J4, J6, J7, J8, J10, J11, J12, J14, J15, J16, J17	13	19.00
Not marked	36-way / 1 row / 2.54 mm pitch straight Pin Header.<br>Schematic parts: J1, J5, J9, J13	1	0.80
Not marked	4.7 KΩ ±5% / 0.25 W / through hole / axial / carbon film resistor.<br>Schematic parts: R1, R2	2	0.24
Not marked	100 µF 50 V ± 20% / Ø 8mm / electrolytic aluminium capacitor<br>Schematic part: C1	1	12.00
Not marked	Printed circuit board fabrication	1	1.50
		**Total Cost**	**23.64**

Arduino firmware

Table S7: Arduino UNO firmware files.

File name.	File type.
LiquidCrystal_I2C-1.1.2	Library
RotaryEncoder-1.1.0	Library
encoder.h	Header
plate.h	Header
screens.h	Header
encoder.cpp	Source
plate.cpp	Source
screens.cpp	Source
fractionCollector.ino	Source

Mounting guidelines

Scheme S1: Expansion board

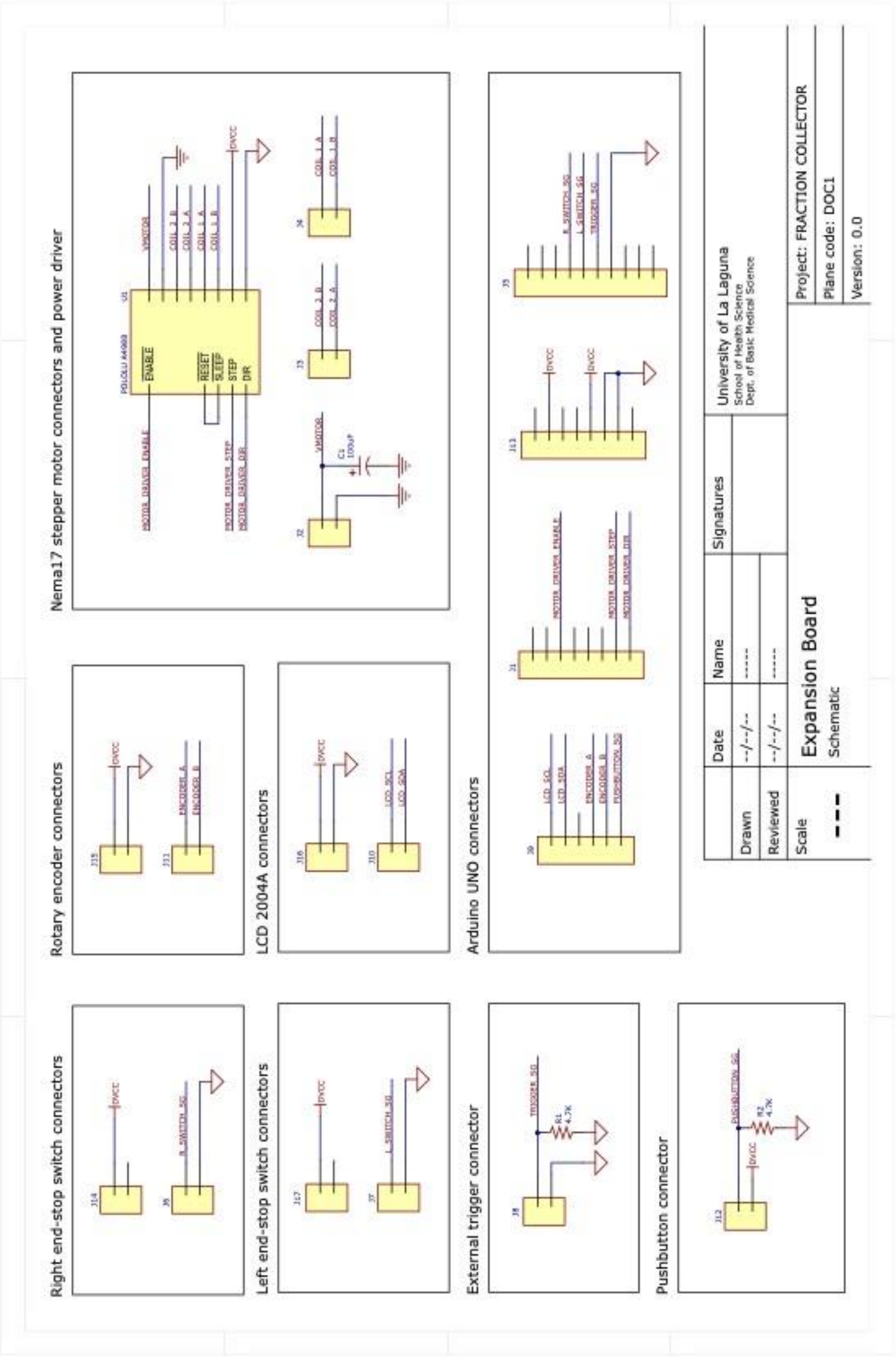

Scheme S2: Expansion board views

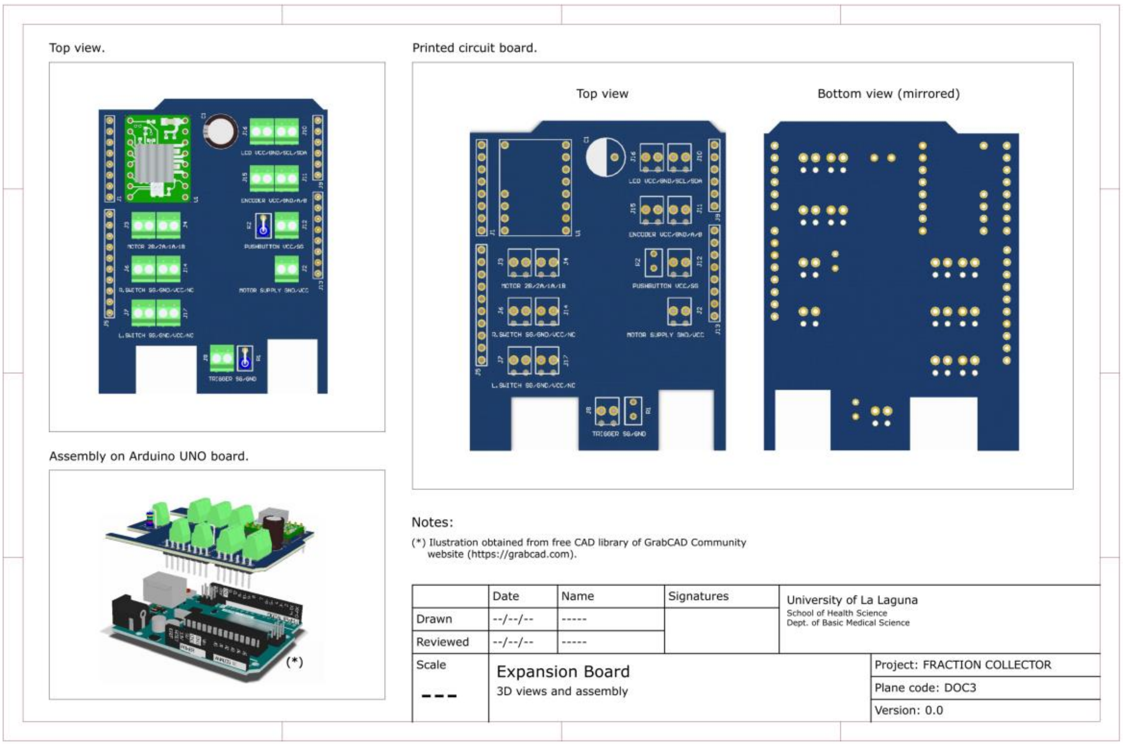

Scheme S3: Wiring

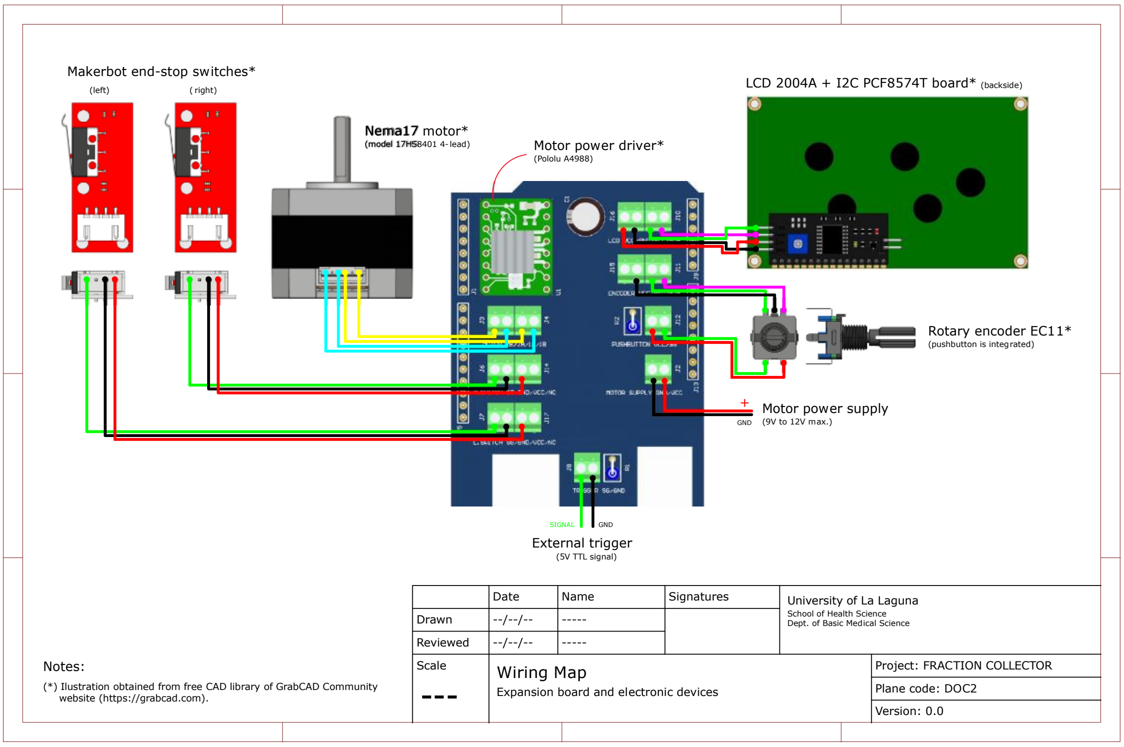

Scheme S4: mechanical assembly

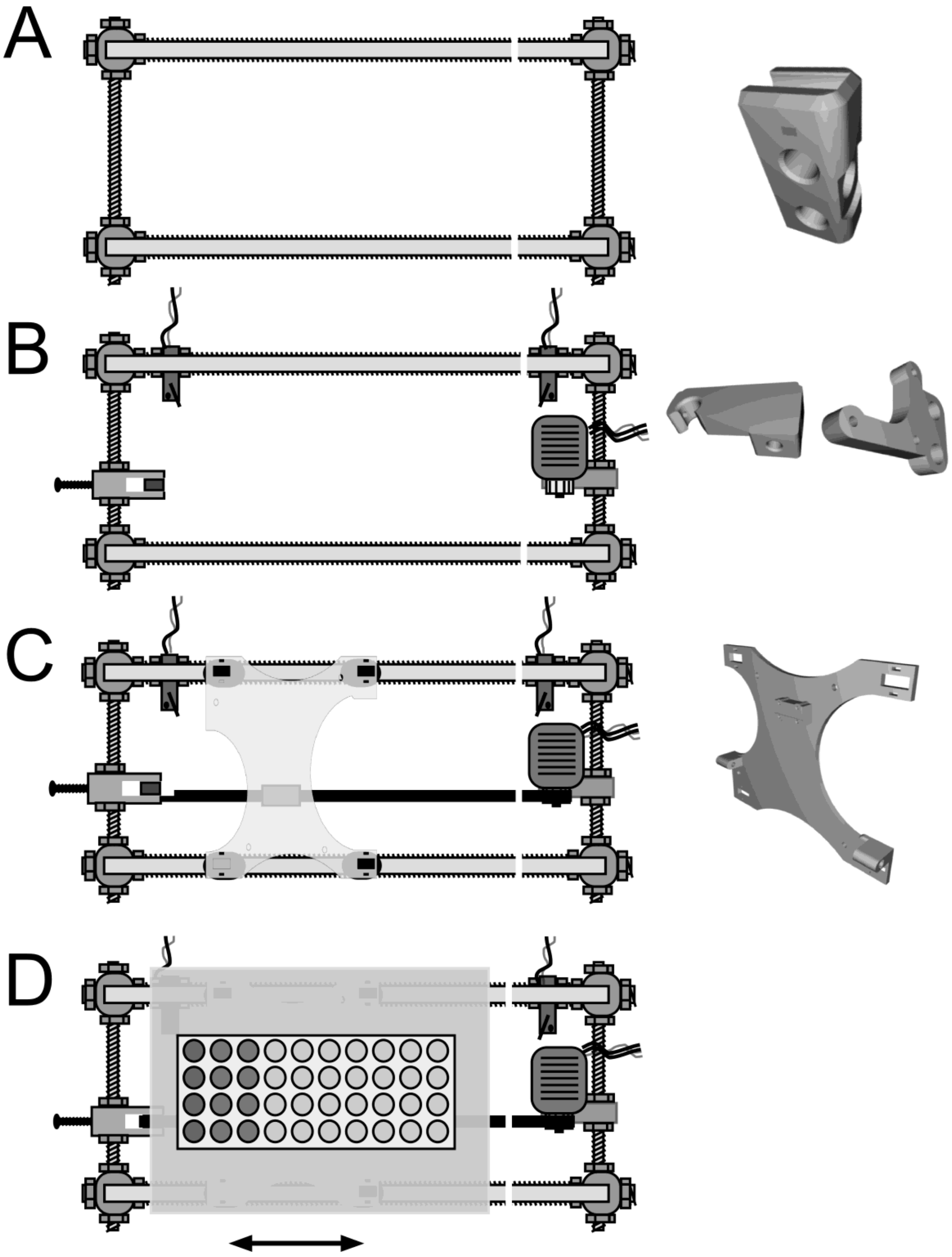

Mounting steps. A, montage of the frame. B, Adding the step motor, the stoppers and the belt tensor. **C,** Adding the belt and the platform with the belt clamp. D, screwing on the methacrylate platform.

Video of the full system working
